# Trait Emotional Intelligence and Classroom Emotions: A Positive Psychology Investigation and Intervention Among Chinese EFL Learners

**DOI:** 10.3389/fpsyg.2019.02453

**Published:** 2019-10-31

**Authors:** Chengchen Li, Jinfen Xu

**Affiliations:** ^1^School of Foreign Languages, Huazhong University of Science and Technology, Wuhan, China; ^2^College of Foreign Languages and Cultures, Xiamen University, Xiamen, China

**Keywords:** positive psychology, trait emotional intelligence, classroom emotions, foreign language enjoyment, foreign language anxiety

## Abstract

The link between emotional intelligence (EI) and negative emotions, especially anxiety, has been investigated in different educational contexts including second/foreign language (L2) learning contexts. However, the link between EI and positive emotions remains underexplored, despite the growing interest of second language acquisition (SLA) researchers in positive emotions, motivated by the Positive Psychology (PP) movement. Grounded on PP theories, a correlational and experimental investigation was conducted on EI and two typical L2 classroom emotions, namely Foreign Language Enjoyment (FLE) and Foreign Language Anxiety (FLA). For the correlational study, questionnaires were administered to 1,718 English learners from three high schools in China. Statistical results showed medium correlations among students’ EI, FLE, and FLA. In the intervention study, a pre-test, treatment and post-test design was adopted. A six-week PP-based EI intervention (“ARGUER” training model in class and the “three activities” of PP in diary) was conducted in the experiment class of 56 students, while not in the control class of 52 students. Semi-structured interviews were conducted with five students in the experimental class and their English teacher. ANCOVA test results and qualitative findings indicated that the EI intervention was effective in improving EI, boosting more positive classroom emotions and alleviating negative classroom emotions. The findings in both the correlational and intervention studies are discussed in combination with previous studies. We also further address their theoretical and practical implications for L2 education.

## Introduction

Positive Psychology (PP) has taken off in the field of Second Language Acquisition (SLA). Its two key commitments are to improve learners’ achievement and to improve their well-being (Li and Dewaele, in press). Emotions and emotional intelligence (EI) constitute the kernel dimension of language learners’ well-being (Oxford, 2016b; Li, 2018). EI was found to be closely related to language anxiety (Dewaele et al., 2008; Shao et al., 2013), empirically supporting the theoretical construct of EI. However, the link between EI and other L2 classroom emotions, especially positive emotions (e.g., enjoyment), remains underexplored. Thus, the present study was primarily designed to investigate the relationship between EI and the two typical L2 classroom emotions (anxiety and enjoyment). Considering the malleability of EI (Mercer and Gkonou, 2017), a post-investigation intervention was also conducted based on the proposed “ARGUER” model and “Three Activities” from PP (see Seligman et al., 2005).

## Literature Review

### Positive Psychology in General Psychology and in Applied Linguistics

The notion of PP has developed immense popularity since Martin Seligman, often called the “father of PP,” was elected as president of the American Psychological Association in 1996. The PP movement, starting with the landmark Special Issue in *American Psychologist* (2000), has catalyzed a shift from clinical psychology’s view of identifying, preventing, and repairing problems, weakness or illness to PP’s view of building strengths in life that contribute to the flourishing of individuals, communities, and societies (Seligman and Csikszentmihalyi, 2000; Maddux, 2002).

Positive psychology is considered to be flourishing with its main topic of “well-being.” Seligman (2011) developed the well-being theory and proposed a five-dimensional PERMA model of well-being, namely “positive emotion (P), engagement (E), relationships (R), meaning (M), and accomplishment (A)” (p. 12). He claimed that the ultimate goal of PP is to improve well-being by increasing the five key areas of “PERMA” including positive emotions. However, PP, with its focus on well-being, does not ignore difficulties, obstacles, and negativity. Instead, it faces them from a strength-based perspective rather than weakness (Snyder and Lopez, 2002). This indicates that PP is not about fixing what is wrong, but about building what is right (Seligman, 2002).

Positive psychology has been flourishing in SLA with its basic commitments of enabling the well-being and L2 success of students (Strzałka, 2016; Szymczak, 2016; Jiang and Li, 2017). Resonating with the “positive renaissance” in general psychology, the area of SLA has also witnessed a “positive turn” (Dewaele and Li, 2018; Li and Dewaele, in press). Lake (2013) may be the first who introduced the term and perspective of PP to the field of SLA (Mercer and MacIntyre, 2014, p. 158). Shortly after the first study linking PP to SLA, the landmark Special Issue on “PP in SLA” came out (2014), showing signs of the advent of the PP movement in SLA. In 2016, two influential anthologies edited by MacIntyre, Gregersen and Mercer, and Gabryś-Barker and Gałajda brought the PP movement to a spurt with diverse topics and rigorous research methods (Al-Hoorie, 2017).

Influenced by Seligman’s well-being theory (2011), PP has largely been mispresented as a way to eliminate obstacles and problems and being pertinent to positive emotions exclusively (Komorowska, 2016). Oxford and Cuéllar (2014), Oxford (2015), and Komorowska (2016) expressed their suspicion of the idealistic approach of creating a difficulty-free learning environment and claimed that language learning is inherently a complex process full of growth as well as obstacles and difficulties, where diverse emotions unavoidably occur, including both positive and negative emotions. Komorowska (2016), and Li (2018) argued that PP in the SLA context is more about seeking value in the obstacles and difficulties of L2 learning and taking a strengths-based approach to them.

In the context of language learning, Oxford (2016a, b) thus modified Seligman (2011) PERMA model of the well-being theory, with an exclusive concentration on positive emotions, to a new theoretical model named EMPATHICS (E: emotional intelligence and emotion; M: meaning and motivation; P: perseverance; A: agency and autonomy; T: time; H: habits of mind; I: intelligence; C: character strengths; S: self-factors). EMPATHICS is an acronym outlining nine complex, interrelated and interacting psychological dimensions. These dimensions are “part of human well-being and positively influence language learners’ achievement and proficiency” (Oxford, 2016a, p. 26). Oxford (2016b) also argued for a holistic look on both positive emotions and negative emotions in L2 learning. According to the EMPATHICS model, the two variables, EI and emotions are primary elements of the first dimension (“E”), both of which are important factors in language learning (Dewaele et al., 2008; Oxford, 2016b, 2018). Furthermore, they are interrelated with each other theoretically. Thus, it is natural to propose the question: **What are the relationships between them empirically?**

### Emotional Intelligence: Constructs, Measurements and Interventions

Before the advent of PP at the very beginning of 21st century (Seligman and Csikszentmihalyi, 2000), EI had already gained extensive attention in general psychology (e.g., Salovey and Mayer, 1990; Goleman, 1995; Mayer and Salovey, 1997; Petrides and Furnham, 2000, 2001). EI has risen to its popularity particularly in the field of PP for its significant role in human performance and well-being, both of which are the primary foci of PP (Allen et al., 2014). EI can therefore be rightfully viewed as a key concept of character strengths within PP (Salovey et al., 2002).

### Theoretical Models of EI

The concept of EI became immensely popular in the media and public, with the release of Goleman’s (1995) influential book entitled *EI: Why It Can Matter More than IQ*. The release occurred long before scientific investigation of its construct and the development of rigorous measurement (Allen et al., 2014). It has been enormously popular especially in organizational psychology. Many organizations started to assess the EI of interviewees as an additional criterion in the absence of psychometrically tested procedures or measurements (Matthews et al., 2002). The urgency to explore the potentially important construct motivated a plethora of definitions, models, and measurements developed by different research groups (e.g., Mayer and Salovey, 1997; Petrides and Furnham, 2000, 2001; Barchard, 2003; Brackett and Mayer, 2003; Bar-On, 2006; Mayer et al., 2016).

Competing theoretical models of EI were classified into two different types: ability models and mixed/trait models (Allen et al., 2014). The ability model conceptualizes EI exclusively as a person’s emotion-related *ability*. The most influential ability construct is the Four-Branch model proposed by Mayer and Salovey (1997). They defined EI within a cognitive-emotional framework as the ability to (1) perceive emotions, (2) use emotions to facilitate thought, (3) understand one’s own emotions as well as those of others, and (4) manage emotions. Task-based measures are required to assess EI as an ability, which remains a challenging task (Li, 2019). The first comprehensive and theory-based task measure for *ability EI* was the Multifactor Emotional Intelligence Scale (MEIS) (Mayer et al., 1999), with its refined successor called the Mayer, Salovey and Caruso Emotional Intelligence Scale (MSCEIT) (2002) (Mayer et al., 2002).

For mixed/trait models, EI was defined as a mix of personality traits including emotion-related personality traits (Petrides et al., 2016). Petrides and Furnham (2000, 2001, 2003) viewed *EI* as a personality trait pertaining to self-perceived emotion-related ability and should thus be measured using self-reported measures. The trait EI questionnaire (TEIQue) (Petrides and Furnham, 2001, 2003) incorporates items not only from the four-branch ability EI model (Mayer and Salovey, 1997), but also from the realm of personality (Goleman, 1995), and intelligences (social, intra-, and inter-personal intelligences) (Gardner, 1983; Goleman, 1995).

Following EI literature in applied linguistics (e.g., Dewaele et al., 2008; Shao et al., 2013; Li, 2019), the present study adopts the trait model of EI. That is, EI is viewed as a personality trait encompassing a cluster of dispositions and self-perceptions related to emotions, positioned at the lower levels of personality hierarchies (Dewaele et al., 2008, 2019b; Alba-Juez and Pérez-González, 2019). Trait EI is measured using self-reported measures including questionnaires (Petrides and Furnham, 2001). This conceptual decision was made to address the following concerns. First, empirical evidence on the very high correlation between EI and personality indicates that EI is substantially a personality trait (Allen et al., 2014). Second, contemporary language learning and teaching have an inherently communicative nature (Mercer and Gkonou, 2017). In other words, collaboration and cooperation prevalently occur among students, and also between language teachers and students. Thus, the construct of trait EI was adopted for its emphasis on and overlap with both intrapersonal and interpersonal EI (Li, 2019). Finally, as mentioned above, task-based tests of ability EI have proven to be a challenging task due to the inherent subjectivity of emotional experience. This indicates that EI is “thus not amenable to truly objective scoring procedures” (Dewaele et al., 2008, p. 919). Trait EI using the self-reported measure was adopted for its practicability, especially in a large-scale study.

Despite conceptual differences, we can conclude a theoretical link between EI and emotions. The link adequately underpins the main research question of the present study, regarding the relationship between EI and L2 classroom emotions.

### EI Studies in SLA: EI and L2 Classroom Emotions

A series of EI-related studies also emerged and confirmed empirically its important role for both language learners (e.g., Dewaele et al., 2008; Shao et al., 2013; Gregersen et al., 2014; Strzałka, 2016; Li, 2019) and teachers (e.g., Gkonou and Mercer, 2017; Mercer and Gkonou, 2017; Dewaele et al., 2018).

As discussed above, EI is a significant predictor of well-being because of its strong link with emotions (Nelis et al., 2011), especially positive emotions. However, the theoretical links are only partially empirically supported in our field. To be more specific, previous studies revealed the negative relationship between EI and negative L2 emotions, particularly, language anxiety. For example, Dewaele et al. (2008) found that adult multilinguals with higher EI tended to feel less anxious. This indicates that individuals with higher EI perceived themselves as more competent in perceiving and understanding the emotions of their interlocutors, managing their own stress and feeling more confident (Oxford, 2017). In a Chinese EFL context, Shao et al. (2013) also found that learners who were more emotionally intelligent generally experienced a lower level of FLA, supporting the link between EI and the negative L2 classroom emotions. However, how EI is related to positive L2 classroom emotions remains to be explored that. Motivated by this gap, the present study is designed to investigate **the links between EI and both positive and negative L2 classroom emotions**, creating a fuller picture of the emotional landscape of language learners.

### EI Interventions Within Positive Psychology

There has been a growing interest in developing school-based interventions aimed at raising EI (Salovey et al., 2002; Miller et al., 2009; Waters, 2011). EI interventions are closely tied to more general social and emotional learning (SEL) programs (Mayer et al., 2002; Brackett and Rivers, 2014), with a focus on developing social and emotional skills, such as emotion regulation and resilience among students, teachers, and school leaders. Its ultimate aim is to foster the well-being and academic achievements of students (Cohen, 1999; Zins et al., 2000; Brackett and Rivers, 2014; Pérez-González and Qualter, 2018).

There are hundreds of curriculum-based SEL programs, most of which provide emotion skill-building opportunities for students to identify, gauge, talk about and to regulate their feelings (Mayer et al., 2002; Brackett and Rivers, 2014). For example, the RULER approach to SEL is a multiyear, structured, comprehensive, and systematic approach that engages all the “stakeholders” involved in students’ education including students, teachers, school leaders, support staff, and family members (Brackett et al., 2009, 2011). The RULER approach is theoretically grounded on the Four-Branch model of Mayer and Salovey (1997) and focuses on developing each stakeholder’s five key emotional skills. Evidence shows that the RULER approach is useful for effective teaching and learning, and promoting well-being and performance for both children and adults (Mayer et al., 2008).

In the field of L2 education, MacIntyre et al. (2019b) argued for PP-based interventions in language learning and teaching to improve “interpersonal relationships, positive emotions, lower stress, greater well-being, and so on” (p. 5). Recognizing the major role of EI in well-being and performance for both language learners and teachers (Oxford, 2016a), Oxford (2016a) argued that there is a need for language teachers to help their students to develop and employ their EI in language learning and contact with others. Mercer and Gkonou (2017) also suggested incorporating training programs to develop EI in order to support effective teaching.

Unfortunately, in SLA, EI-specific interventions based on PP principles remain at a starting stage. There are quite a few theoretically grounded and evidence-based intervention practices whose efficacy have been tested. Among the limited number of attempts, Gregersen et al. (2014) innovatively implemented EI interventions to an English learner and a pre-service TESOL teacher by adapting three activities from PP whose efficacy have been empirically tested. The three activities include identifying “three good things” (see Seligman et al., 2005), “savoring” positive experiences (see Peterson, 2006) and developing participants’ “learned optimism” in adverse experiences related to language teaching, learning, or education (see Seligman, 2006) (Gregersen et al., 2014, p. 340–347). However, as pointed out by Gregersen et al. (2014), the intervention practice could be more EI-oriented with exercise tightly tied to specific emotional skills, which may bring greater outcomes. Furthermore, a qualitative approach was adopted to focus on intervention effectiveness, leaving room for other research methods which can better visualize the changes before and after intervention practices.

Thus, **the EI intervention implemented in the present study was designed to improve EI-specific skills based on the theoretical constructs of *trait EI***. A mixed method of both quantitative and qualitative approaches was adopted to test the efficacy of EI interventions for language learners.

### L2 Classroom Emotions

Emotion had traditionally been neglected as “irrational factors” until the introduction of humanistic language teaching values in the 1970s and 1980s. Scholars began to take a holistic view on language learners, emphasizing their cognition and emotion equally (Arnold, 1998, 1999; Al-Hoorie, 2017; Piniel and Albert, 2018; Dewaele et al., 2019a this volume). Echoing this view, the *Affective Filter Hypothesis* (Krashen, 1985) was proposed to underline the fundamental role of emotion in language learning. The hypothesis motivated a plethora of studies on language anxiety in the past three decades (Gkonou et al., 2017).

However, the advent of PP in SLA (MacIntyre and Gregersen, 2012; Mercer and MacIntyre, 2014) catalyzed an “affective turn” and a “positive movement.” The broaden-and-build theory of positive emotions (Fredrickson, 1998, 2001, 2003) underpinned the emotional movement in SLA. This has contributed to a shift from an exclusive focus on anxiety to a wide range of classroom emotions including both positive and negative emotions (Dewaele and Li, 2018; MacIntyre et al., 2019b; Prior, 2019). A great variety of different L2 classroom emotions other than anxiety have been reported by language learners and users in different contexts. Pishghadam et al. (2016), for instance, investigated the emotional experiences of 308 Iranian EFL university students and found that positive and negative classroom emotions coexisted with each other. The major positive foreign language emotions identified were “enjoyment, pride, and hope, and the major negative emotions were anxiety, shame, boredom and helplessness” (p. 508). Galmiche (2017), and Ross and Stracke (2017) studied the emotional experiences of shame and pride in the language learning process. In the study of MacIntyre and Vincze (2017), situated within a German-as-a-foreign-language context of Italian language secondary schools, 10 positive emotions including “joy, gratitude, serenity, interest, hope, pride, amusement, inspiration, awe, and love” (p. 61) and nine negative emotions including “anger, contempt, disgust, embarrassment, guilt, hate, sadness, feeling scared, and being stressed”(p. 61) were identified as basic L2 motivation-related emotions. MacIntyre et al. (2019a) further investigated an inclusive but not exhaustive list of ten positive emotions (“interest, excitement, strength, enthusiasm, pride, alertness, inspiration, determination, attention, and activeness”) (p. 4 in the text) and ten negative emotions (“distress, upset, guilt, scare, nervousness, hostility, irritation, shame, jitter, and afraidness”) (p. 4 in the text) evoked over the past week among L2 learners.

Furthermore, in the first two Special Issues on L2 emotions, namely “Emotions in SLA” and “A PP perspective on emotions in SLA” (Li, in press), a variety of emotions were examined among learners of distinctive language profiles (diverse first languages as well as target L2s) at different instruction levels (from primary level to tertiary level) in diverse L2 learning contexts (countries all over five different continents). For example, Pavelescu and Petrić (2018) investigated the emotional experiences of four Romanian EFL high school students and found that enjoyment and love were the most frequently experienced positive emotions during their language learning process. Pavelescu and Petrić (2018) explored the emotion types that Hungarian English majors experienced in their general use of the L2 and four specific language skills. Nine in- and out-of-class emotions were identified. Seven positive emotions were identified, namely “pride, contentment, comfort, relaxation, and enjoyment,” and two negative emotions were identified, namely “anxiety and not like” (p. 142).

The fruitful findings on diverse classroom emotions does not mean that emotions or positive emotions had not been investigated before 2012, in fact, many researchers and practitioners laid the foundation for the “affective turn” within PP (e.g., Arnold, 1999; Cook, 2000; Broner and Tarone, 2001; Brantmeier, 2003, 2005; Kramsch, 2006; Imai, 2010). However, these studies are flourishing with the PP movement in SLA, creating the strong wave of scholarly interest in L2 classroom emotions that we are currently witnessing (Dewaele and Li, 2018; White, 2018).

Despite the growing number of studies in emotions flourishing with PP movement in SLA, in the Chinese EFL context, studies on emotions are still greatly confined to anxiety, with PP at its starting stage (Li and Dewaele, in press).

### Foreign Language Enjoyment and Anxiety

In the above-mentioned list, enjoyment and anxiety were reported as two of the most prevalent classroom emotions. They were metaphorically conceptualized as “malevolent and benevolent wolves” (Gregersen et al., 2017) and “feet” (Dewaele and MacIntyre, 2016) for every language learner despite their language proficiency. This could also manifest as a result of the large number of studies on the two emotions termed as Foreign Language Enjoyment (FLE) and Foreign Language Anxiety (FLA) in diverse contexts of L2 learning from a PP perspective (e.g., Dewaele and MacIntyre, 2014, 2016; Dewaele and Dewaele, 2017; Dewey et al., 2018; Elahi Shirvan and Taherian, 2018; Khajavy et al., 2018; Jiang and Dewaele, 2019; Li et al., 2019).

Previous studies have consistently confirmed the triangular relationship between FLE, FLA and L2 achievement. More specifically, there is a significant positive relationship between FLE and L2 achievement, while negative relationships between FLA and L2 achievement, and between FLE and FLA (e.g., Dewaele and Alfawzan, 2018; Jin and Zhang, 2018; Saito et al., 2018; Li et al., 2019). This indicates that learners who enjoy more in their L2 exploration tend to feel less anxious and achieve more. In terms of the correlation between FLE and FLA, despite the general negative relationship pattern between them, Dewaele and MacIntyre (2016) also pointed out that they are two separate emotions instead of two poles of a same dimension and that other interacting patterns exist between them.

Previous studies have also revealed a large list of correlates of the two emotions, including learners’ socio-psychological-behavioral variables and teacher-centered variables (e.g., Dewaele and MacIntyre, 2014; Saito et al., 2018; Dewaele et al., 2019c). Among them, EI was revealed as a significant predictor of language anxiety (Dewaele et al., 2008; Shao et al., 2013). However, the link between EI and positive L2 classroom emotions including FLE remains underexplored (Li, 2019). Furthermore, previous studies exclusively used a quantitative method to reveal the links between EI and FLA, inadequately showing exactly how EI is used by L2 learners to cope with emotion-related information in their L2 learning. Thus, in the present study, a quantitative approach was adopted to investigate the relationship between EI and the two prevalent classroom emotions of FLE and FLA, supported by a qualitative approach showing in detail the employment of EI in different positive and negative emotional experiences in English learning.

## Research Questions

Motivated by the gaps exhibited in previous studies, the following research questions were formulated. Specifically, Study 1 was guided by RQs1-2, and Study 2 was an intervention practice guided by RQ3.

RQ1: What are the levels of students’ EI and English classroom emotions?
RQ2: What are the interrelationships between students’ EI and English classroom emotions?
RQ3: Is the ARGUE training model of EI effective to boost more positive emotions and alleviate negative classroom emotions?

## Study 1: Investigation of Trait Emotional Intelligence and Classroom Emotions

### Methods

The present study followed an exploratory sequential design. It first reveals statistically significant patterns quantitatively, and then validates, enriches, and embellishes the quantitative results with qualitative findings (Creswell and Clark, 2011, p. 81).

#### Participants and Local Context

The participants for the present study were 1,718 second-year senior high school students from three schools in Lu’an City, China. This suggests that they followed the same curriculum for the English course regulated by the Ministry of Education of the People’s Republic of China and meant that they were at same instruction level. They were going to take the National College Entrance Examination in their third academic year. A standardized English test is part of this entrance exam, a prerequisite for admission to universities. Furthermore, for all the participants, Chinese (Lu’an dialect or Mandarin) was their L1 and English was their only FL. None of them had overseas experience. Furthermore, reckoning with previous findings that students at different proficiency levels may show different psychological characteristics (Saito and Samimy, 1996; Marcos- Llinás and Garau, 2009; Dewaele and MacIntyre, 2014), the participants involved were from three schools of different types and at varying academic levels (i.e., two public schools and one private school, and from the Top 1 school to schools of lower rankings according to the local academic ranking in Lu’an City).

Detailed information of the participants is displayed in Table 1.

**TABLE 1:** Participants’ demographic information (N = 1,718).

	School	No.	Male	Female	HSS	NS	Mean Age (SD)
Quantitative	A	349	195	154	38	311	16.61 (0.75)
Phase	B	439	253	186	186	253	16.69 (0.75)
(EI, FLE and FLCA)	C	930	447	483	743	187	16.93 (0.75)
	Tot.	1,718	895	823	967	751	16.81 (0.77)
Qualitative phase (EI)	C	64	35	29	20	44	17.74 (0.64)

*HSS = Humanities and social sciences; NS = natural science.*

#### Instruments

##### Trait emotional intelligence questionnaire – short form (TEIQue–SF)

The TEIQue instruments were developed on the basis of *trait EI* theory (Petrides et al., 2007; Petrides, 2010). The TEIQue–SF is the short version (consisting of 30 items, two items for each of the 15 facets) of the full-form TEIQue (comprised of 153 items) (Petrides and Furnham, 2006; Petrides, 2009). Two items were selected for each of the 15 facets from the full-form TEIQUE and included in the TEIQue–SF. The selection was primarily based on the correlations between scores for each item and for the total facet. Both forms of TEIQue adopt a 7-point Likert-type response scale, ranging from “1 (Disagree completely)” to “7 (Agree completely),” suggesting that the possible score of participants range from 30 to 210. Correspondingly, total scores below 120 signify an underdeveloped EI; total scores between 120 and 150 indicate a moderately developed EI; and total scores above 150 denote a well-developed EI.

The reliability (Cronbach’s alpha) of the short form proves to be very satisfactory in several studies in different FL contexts (e.g., Cronbach’s alpha = 0.79, *N* = 425; Dewaele et al., 2008) including the Chinese EFL context (Cronbach’s alpha = 0.86, *N* = 510; Shao et al., 2013), usually higher than 0.80 and has not dropped below 0.70 in any study. The alpha for the scale in the present study was found to be as high as 0.796 (*N* = 1,718).

Previous studies also showed that the measure had a relatively stable construct validity across languages (Mavroveli et al., 2007; Mikolajczak et al., 2007; Andrei et al., 2016). The four factors that are confirmed are 0.74 (Emotionality), 0.76 (Self-Control), 0.80 (Sociability), and 0.85 (Well-Being), respectively (Petrides, 2009), with 15 subscales (e.g., emotion perception, emotion expression, emotion management, social awareness, trait optimism, self-motivation, empathy, etc.; for a full list, see Petrides, 2009, p. 89, 93).

Following the study of Shao et al. (2013) in the EFL context in China, the present study also adopted the translated version of the TEIQue-SF. The original TEIQue-SF was translated into Chinese by the first author and further checked by three professionals in the field of applied linguistics, English literature, and psychology, respectively.

##### Chinese version of foreign language enjoyment scale

The FLE Scale was originally developed using an international sample, with a majority (67.2%: 1171/1742) of European participants (Dewaele et al., 2016, p. 245). In the Chinese EFL context, Li et al. (2018) translated the 14-item and two-factor *FLE Scale* (Dewaele and MacIntyre, 2016) into a Chinese one, and modified it using a Chinese sample (*N* = 1,718). The modified *Chinese version of Foreign Language Enjoyment Scale (CFLES). The CFLES turned out* to have eleven items with a new three-factor structure confirmed (i.e., *FLE-Private*, *FLE-Teacher* and F*LE-Atmosphere*, Li et al., 2018). The CFLES adopts a 5-point Likert scale, ranging from ‘1 (Strongly disagree)’ to “5 (Strongly agree),” with possible scores ranging between 11 and 55. Within the range, a total score below 33 indicates low or no FLE, a total score between 33 and 44 denotes a moderate level of FLE; and a total score above 44 signifies a high level of FLE.

Li et al. (2018) (*N* = 1,718) reported a high reliability for the global CFLES and its subscales of *FLE-Private*, *FLE-Teacher*, and *FLE-Atmosphere*: 0.826, 0.792, 0.896, and 0.778, respectively. The split-half reliability was reported to be as high as 0.878. Strong construct validity (CFA) [*χ^2^*(41) = 72.975; CFI = 0.975; TLI = 0.967; SRMR = 0.034; RMSEA = 0.041], convergent validity, and discriminant validity were also found in the study of Li et al., 2018, p. 188–190). The present study was based on the same dataset as Li et al. (2018), indicating the same reliability and validity for FLE.

##### Foreign language classroom anxiety scale

*The Foreign Language Classroom Anxiety Scale (FLCAS)* was originally designed to measure anxiety specific to foreign language learning contexts (Horwitz et al., 1986). It is a well-established instrument that has been widely applied in different countries with learners of various L2s and L1s (e.g., Aida, 1994; MacIntyre et al., 1997; Matsuda and Gobel, 2004; Marcos- Llinás and Garau, 2009; Park and French, 2013; Shao et al., 2013). It is comprised of 33 items, responded to on a 5-point Likert scale, ranging from “1 (Strongly agree)” to “5 (Strongly disagree).” It is generally recognized as having a one-factor structure (Horwitz et al., 1991) concerning performance evaluation within both academic and social contexts.

It demonstrated very high internal reliability (α = 0.93, *n*≈300) and high test-retest reliability with an interval of 8 weeks (*r* = 0.83, *p* < 0.001, *n* = 78) in the FL context of the U.S., with all items significantly correlated to a global scale (Horwitz, 1986, p. 560).

High validity of the scale has also been demonstrated in criterion-related studies, specifically by investigating the correlation of the FLCAS with other closely related scales such as with Spielberger (1983) *Trait scale of the State-Trait Anxiety Inventory* (*r* = 0.29, *p* < 0.01, *n* = 10), with Watson and Friend (1969) *Fear of Negative Evaluation Scale* (*r* = 0.36, *p* < 0.01, *n* = 56), and with Sarason (1978) *Test Anxiety Scale* (*r* = 0.53, *p* = 0.001, *n* = 60) (Horwitz, 1986, p. 560, 561). In terms of the construct validity, FLCAS is generally considered as having a one-factor structure with three related kinds of anxiety integrated, i.e., communication apprehension, test anxiety, and fear of negative evaluation (Horwitz et al., 1986, 1991; Horwitz, 2017).

The present study adopted the Chinese version of FLCAS validated by Wang (2003). In the present study, the Cronbach’s Alpha for the FLCAS is 0.920 (*N* = 1,718), showing that the scale has high reliability.

#### Open Questions for Emotional Intelligence and Classroom Emotions

Two open questions were designed for the participants to elicit data on EI and classroom emotions. Based on the trait emotional intelligence theory (Petrides et al., 2007; Petrides, 2010), the EI-related questions in L2 classroom settings were designed as follows: (1) “Do you have awareness of your feelings in L2 learning? (2) Do you have awareness to regulate, manage or control them, and if so, how do you do that?”

#### Procedures

There were two phases involved in data collection, a quantitative phase and a qualitative phase, respectively. In the quantitative phase (from early May to June 2017), a composite questionnaire was adopted to retrieve data for participants’ demographical information (e.g., gender, age, and academic discipline), as well as the test variables in the present study including TEI, FLE, and FLA. Before the administration of the questionnaires, both written and oral consent was obtained from different sides including school presidents, headmasters and English teachers as well as the students from each school. For the 49 students under 16 years old, written consent was also obtained from their guardians (parents or grandparents). Paper-and-pen questionnaires in a classroom situation were adopted because of the prevalent ban of mobile phones and personal computers in most schools at primary and secondary levels in China. In both oral and written instructions, students were assured that all the information they provide would remain confidential, and that all the data would be analyzed and used only for research purposes in an anonymous way. Furthermore, absence in the study or withdrawal from the study at any time would have no consequence for the students.

The qualitative stage took place in October 2017. Participants were involved and asked to respond to two open questions.

### Quantitative Results and Discussion

The following findings were obtained in response to the research questions.

#### Levels of Emotional Intelligence and L2 Learning Emotions

Before doing referential analyses, Kurtosis and Skewness were calculated to establish that EI, FLE, and FLA exhibited normality (see Table 2). Descriptive statistics was then conducted to provide a general profile of students’ EI, and two typical classroom emotions of FLE, and FLA. Table 2 displays the results for the test of normality (skewness and kurtosis with their standard errors), and the parameters of mean score, standard deviation, median, mode, minimum and maximum for these target variables.

**TABLE 2:** Descriptive statistics for emotional intelligence and classroom emotions (*N* = 1,718).

**Variable**	**Score range**	**Mean**	***SD***	**Mdn**	**Mode**	**Min**	**Max**	**Skewness**	***SE1***	**Kurtosis**	***SE2***
EI	30–210	134.09	17.87	135	130	54	198	–0.07	0.06	0.52	0.12
FLE	11–55	34.35	6.96	34	34	11	55	–0.29	0.06	0.66	0.12
FLA	33–165	100.19	19.87	101	103	35	164	–0.05	0.06	0.19	0.12

*EI = Emotional intelligence; FLE = Foreign language enjoyment; FLA = Foreign language classroom anxiety.*

Trait emotional intelligence questionnaire – short form has 30 items coded on a 7-point Likert scale, indicating a possible score range between 30 to 210. As exhibited in Table 2, all of the three descriptive parameters of EI, namely mean score, median, and mode fell into the middle-to-high range of 120–150. The mean scores of the EFL learners in the present study were lower than those in previous studies at Chinese university levels (*M* = 140.78, *SD* = 20.75; Shao et al., 2013). This difference may be explained by the claim that EI may improve with age (Salovey and Mayer, 1990) and be developed through experience (Mercer and Gkonou, 2017). Additional frequency analysis showed that 5 (0.29%), 518 (30.15%), 1,136 (66.12%), and 59 (3.43%) out of the 1,718 participants fell into the low, low-to-middle, middle-to-high, and high range of EI, respectively, indicating the general tendency of most students (about 70%) to perceive themselves as moderately to highly emotionally competent in coping with emotion-laden information of their own as well as others. Standard deviations (17.87) in Table 2 indicate great variations among students’ EI scores. The Max. (198) and Min. (54) indicated that some students reported very high EI, while others reported a very low level of EI, highlighting the huge individual differences among the participants. This suggests that EFL teachers should be aware of and psychologically prepared for the inherent differences in emotion-related abilities among students, especially in FL classrooms which are infused with various emotional experiences arising from interactions between the FL teacher and students (Miyahara, 2015).

The CFLES is a 5-point Likert scale with 11 items, and thus participant scores range between 11 to 55. As shown in Table 2, all three parameters of mean, median, and mode fell between the middle-to-high range of 33–44; however, they were just slightly higher than the threshold score of 33, indicating that participants experience a moderate level of FLE. An additional frequency analysis showed that 84 (4.89%), 656 (38.18%), 873 (50.81%) and 105 (6.11%) out of the 1,718 participants fell into the low, low-to-middle, middle-to-high, and high range of FLE, respectively, indicating that a large percentage (more than 40%) of participants reported experiencing low or little FLE. Standard deviations (6.96) in Table 2 indicate great variations among the FLE scores of the participants. The Max. (55) and Min. (11) indicated that some students reported experiencing high levels of enjoyment, while others reported experiencing little or no enjoyment in English learning, pointing to the inter-individual variability among the learners in the emotional experiences of enjoyment in English learning.

The FLCAS has 33 items coded on a 5-point Likert scale, with a possible score range between 33 to 165. According to the mean, median, and mode displayed in Table 2, participants reported a middle-to-high level of FLA, indicating the general tendency that most participants experienced moderate levels of FLA. Additional frequency analysis showed that 81 (4.71%), 733 (42.67%), 817 (47.56%), and 87 (5.06%) out of the 1,718 participants fell into the low, low-to-middle, middle-to-high, and high range of FLA, respectively, indicating that a large percentage (52.62%) of participants reported experiencing a middle-to-high or high level of FLA. Standard deviations (19.87) in Table 2 indicate great variations among the FLA scores of the participants. The Max. (164) and Min. (35) indicated that some students reported experiencing a high level of anxiety, while others reported experiencing a low level of anxiety, pointing to the huge individual differences among students in their experiences of anxiety in English learning.

An independent-samples *t-test* was conducted manually on the average score for each item in the FLE scale as well as the FLA scale between the Chinese sample in the present study and the international sample in the study by Dewaele and MacIntyre (2014). The results (see Table 3) shows that participants in the present Chinese EFL context reported a significantly higher level of FLA (*t* = −3.195, *p* < 0.01, *d* = −1.27, *r* = −0.54), while lower levels of FLE *(t* = 11.79, *p* < 0.001, *d* = 0.40, *r* = 0.20) were reported compared to their counterparts in the international sample. This also echoes the research findings that Chinese L2 learners scored relatively low in positive emotions and high in negative emotions in their L2 exploration (MacIntyre et al., to appear).

**TABLE 3:** Comparisons of classroom emotions between the present study and the study by Dewaele and MacIntyre (2014).

**Studies**	**Contexts**	**FLE**		**FLA**	
			
		**Mean (range)**	***SD***	**Mean (range)**	***SD***
The present study	China (*N* = 1,718)	3.12	0.63	3.04	0.60
Dewaele and MacIntyre (2014)	International (*N* = 1,742)	3.82	0.46	2.75	0.83

One possible reason could be the very strong exam orientation in FL learning in China and the high stakes it represents. Many students take extra English classes in ‘cram schools,’ from pre-school to the tertiary level, which can increase their levels of language-related stress. Compared to students in other parts of the world, Chinese students endure a considerable degree of cultural pressure: “being raised in a collectivistic culture which places a high value on education and filial piety, Chinese adolescents are pressured to achieve academically, not to disappoint their significant others, and to maintain their social identity” (Essau et al., 2008; p. 803). Although the exact cause of these great differences remains to be explored, considering the relatively high level of FLA and low level of FLE, we can safely conclude that there is considerable room for improvement in English education in a Chinese senior high school context. In fact, English classes are required for senior high school students almost each weekday, it is thus troubling to see students learning English with little or no enjoyment but with a lot of anxiety. Despite the distinctive effects of positive and negative emotions in L2 achievement, we suggest taking a holistic view within PP on leaners’ development and investing more efforts in maintaining and improving students’ emotional well-being, for instance, by fostering more positive emotions like enjoyment and alleviating the effects of negative emotions like anxiety.

#### Correlations Among Students’ Emotional Intelligence and Classroom Emotions

Pearson correlation analyses were conducted among the three variables after the above-mentioned normality test (Table 2).

Medium sized correlations (Plonsky and Oswald, 2014) were found between each two variables (see Table 4). To be more specific, students’ TEI was significantly related to both classroom emotions, in a positive relation to FLE while in a negative relation to FLA, indicating that more emotionally intelligent English learners tended to enjoy more and feel less anxious during their language exploration. This provides insightful pedagogical implications in that the improvement of TEI may contribute to more positive emotional experiences and fewer negative emotional experiences. Thus, training or intervention of TEI may also further function as intervention on classroom emotions including FLE and FLA. Qualitative analyses in the subsequent section will shed more light on the EI and L2 learning emotions of students.

**TABLE 4:** Correlations among students’ emotional intelligence and L2 classroom emotions.

	**1**	**2**	**3**
TEI	–		
FLE	0.313^∗∗∗^	–	
FLA	–0.389^∗∗∗^	–0.438^∗∗∗^	–

**N* = 1,718. ^∗∗∗^Indicates statistical significance at a *p* < 0.001.*

Consistent with previous studies (e.g., Dewaele and MacIntyre, 2014, 2016; Dewaele and Alfawzan, 2018; Li et al., 2019), the present study revealed a significant negative relationship between the two prevalent classroom emotions. This suggests that in general leaners experiencing more enjoyment tended to feel less anxious in their L2 learning, and *vice versa*. This also suggests that boosting more enjoyment may be employed as an intervention to distracting learners from their “anxious selves.” A qualitative study shows more details of learners.

### Qualitative Findings

#### Emotional Intelligence and L2 Classroom Emotions

Fifty-five participants (15 males, 40 females) answered the open question related to EI. These questions were organized based on the theoretical constructs of EI (Petrides, 2009). More specifically, participants were asked a set of questions related to their EI. More specifically, they were asked whether they had awareness of their feelings during L2 learning, and whether they had the awareness to regulate, manage, or control them, and if so, how to do that.

Eleven out of the 55 participants reported that they had no awareness of their feelings in L2 learning while the remaining 44 participants reported that they had a strong awareness of their feelings and emotions in L2 learning. Furthermore, they drew on their TEI to cope with the feelings and emotions they experienced.

The narratives were analyzed and salient themes were identified when they support, complete, or enrich the quantitative findings on the relationship between TEI and positive and negative L2 learning emotions. In other words, they were coded when they reflected participants’ ability to effectively respond to and cope with an emotional experience in their language journey.

The narratives showed that most participants were aware of their feelings especially in stressful situations or when experiencing anxiety. They also had the awareness of the necessity to cope with negative emotions (e.g., anxiety, stress). Specifically, first, some of them tended to make themselves *feel more neutral* by self-distraction, engaging themselves in something or some events unrelated to the present emotional stimulus. For example, some students responded as follows, “When I felt so stressed or anxious in English learning, I would just do something unrelated to English, requiring no attention or cognitive efforts.” Strategies including reading non-English books, listening to music, sleeping, and having a cup of coffee were frequently mentioned as effective ways to distract themselves from the stressful or anxious situation. Second, some students also reported that they tried to *make themselves feel more positive or opposite emotions* in stressful or anxious situations by distracting themselves with something which can boost positivity. For example, they mentioned involving themselves in interesting English songs which may enhance their interest and likings toward English. They also mentioned thinking of things in English learning which brings happiness and motivates them to learn. They emphasized thinking positively in emotional regulation, especially changing their mood, and boosting their confidence in English learning by having clear and attainable goals.

Besides the self-distraction, some students reported *self-comforting experiences* in stressful and anxious situations. Taking a breath, closing ones eyes, and trying to calm down were frequently mentioned self-soothing behaviors.

Unlike self-distraction and self-comforting, other students reported their experiences of *employing the psychological process of motivation and cognition* to cope with their negative feels or emotions such as anxiety, frustration, and stress directly. For example, some students reported that when they felt anxious or stressed, they would make more efforts and be fully attentive in ongoing tough tasks (e.g., reading, recitation, English class), which was originally the negative emotional stimulus. Other students reported that when they felt frustrated or stressed, they would remind themselves of the great importance of English and the benefits of having a good command of English to make themselves feel more extrinsically motivated in English learning.

Altogether, considering the findings, we can conclude that they were closely tied to the theoretical constructs of TEI (Petrides, 2009, p. 89). For example, the findings empirically instantiate the facets of TEI in the specific domain or context of EFL learning. For example, the following facets including “emotion perception,” “emotion regulation,” “stress,” “self-esteem,” “trait happiness,” “trait optimism,” “adaptability,” and “self-motivation” (Petrides, 2009, p. 89) were found in the narratives. To conclude, TEI is related to the emotional awareness, as well as the ability to respond and cope with negative emotions, or to generate more thought-facilitative positive or neutral emotions, supporting the quantitative findings on the significant relationships between TEI and two typical kinds of emotions in L2 learning. Thus, we can argue that, students who have a higher level of TEI tended to feel less anxious and enjoyed more in a certain situation *via* specific regulatory strategies.

### Conclusions and Implications

The quantitative and qualitative results converge to confirm the significant relationships between TEI and both positive and negative L2 classroom emotions, and the general negative relationship between FLE and FLA with other interacting patterns.

The present study shows its main limitation in its measurement. The quantitative data was retrieved only from self-reported scales, meaning that the emotional abilities in the present study are basically self-perceived emotion-related abilities and are unavoidably amenable to social desirability bias. Furthermore, the traditional research methods based on retrospective data collection may either overlook the short-lived and dynamic feature of emotions in the astatic and fluctuating communication process, or define an emotion partially as one specific emotional reaction, which is significantly stronger or occurs closer to the end of the event.

Despite the limitation, the present study still provides insightful theoretical and practical implications. Theoretically, the quantitative and qualitative findings converge to support the theoretical construct of TEI, linking TEI to both positive and negative emotions empirically in our field of L2 learning. Practically, the quantitative and qualitative findings highlight the co-existence of a great variety of L2 classroom emotions. This provides pedagogical implications that the teacher should be psychologically aware of and should be prepared to embrace leaners’ affective ambivalence (MacIntyre et al., 2009) instead of attempting to create an ideal negativity-free learning environment for their students. Instead, it is more fundamental to equip students with relatively stable emotional strengths and emotional ability to cope with situation-dependent, short-lived and dynamic emotions.

The findings also show the relationships between TEI and different L2 classroom emotions, complemented by qualitative findings on how EI was employed to cope with emotions. Grounded on both the empirical findings and the theoretical links between TEI and emotions, the following intervention of EI was implemented to improve students’ EI and further affect their classroom emotional experiences.

## Study 2: Ei Intervention Based on the “Arguer” Model and “Three Activities”

### Methods

The whole intervention program is summarized in Table 5 and subsequently described in greater detail. The outline for the training is presented in the Appendix.

**TABLE 5:** Summary of the intervention practice.

**Procedures**	**Activities**	**Participants**		**Instruments**	**Duration/frequency**
				
		**Intervention**	**Control**		
Pre-intervention survey	Questionnaire 1	56 students	52 students	*TEIQue–SF* CFLES FLCAS	Once
EI intervention	EI training	56 students	/	ARGUER	6 h (1 h per week)
	Diary	56 students	/	ARGUER and “Three Activities” from PP	12 times (twice per week)
Post-intervention survey	Questionnaire 2	56 students	52 students	*TEIQue–SF* CFLES FLCAS	Once
	Interview	5 students and their English teacher	/	Face-to-face semi-structured interview	Once

#### Participants

Two classes from a boarding high school (School D) in Lu’an City, China, were involved in the whole program. In this school, most of the students were students who were left-behind, without their parents living with them. The two participating classes were randomly assigned as the intervention group and the control group, respectively. They had the same English teacher. For the intervention group, 56 students (Mean age: 16.12; *SD*: 0.74) of Year One participated in the pre- and post- intervention surveys and the intervention activities (“ARGUER” approach to EI training and diary reflection). Five of the students and their English teacher took the post-intervention interview. In the control group of 52 students (Mean age: 16.22; *SD*: 0.73), there was no intervention practice and the students were only required to complete the pre- and post- intervention questionnaires. Before the conduction of the intervention program, we obtained both written and oral consent from the principal of the school, the headteachers of the classes, the English teacher, and the students. We also obtained written consent from 11 guardians (parents or grandparents) of those students under 16 years old.

#### Instruments

##### Composite questionnaire

The instruments used for pre- and post- intervention questionnaire surveys were composed of TEIQue–SF (Petrides and Furnham, 2006; Petrides, 2009), CFLES (Li et al., 2018), and FLCAS (Horwitz et al., 1986) (refer to the section of Instruments in the previous large-scale investigation study).

##### The “ARGUER” model

ARGUER model of EI training was based on *trait EI theory* (Petrides and Furnham, 2000, 2001, 2003) and *four-branch ability model of EI* (Mayer and Salovey, 1997) and designed by adapting the RULER model (Brackett et al., 2009, 2011). The ARGUER model was designed mainly in combination with emotional experiences in EFL learning/use-specific contexts. The acronym represents six interrelated emotional skills, indicating that the acronym here is not intended for taxonomy or hierarchy, instead each skill is likely to influence another. The first author was the trainer, conducting the training of all the six “ARGUER” dimensions throughout the 6 weeks (1 h for one EI dimension training in 1 week). Herein, for simplicity, we describe briefly each of them separately.

##### Awareness of feelings and emotions in self and others in week 1

An inductive approach was adopted throughout the training program. First, the first author, the trainer in the intervention program, asked the students and the English teacher to discuss the importance of emotions in different life aspects including English learning and teaching supported by their own experiences. Then, they were asked to reflect on the degree of their awareness of the emotions in themselves and others in general contexts as well as in English learning and teaching contexts. Finally, the trainer made a summary by emphasizing the importance of emotions, and awareness of emotions, and showing the necessity of developing one’s emotional awareness as a habit both in daily communication and English learning and teaching.

##### Recognizing emotions in week 2

The students were asked to reflect on their ability to recognize the emotions in others including their classmates and English teacher. Verbal and non-verbal cues were mentioned as basic ways for them to recognize their English teacher’s emotions. They were also asked to support themselves by using examples in the English class (the English teacher was informed of the potential discussion related to her beforehand). Comparatively speaking, the emotions of their classmates were seldomly discussed. Instead, they mentioned classroom atmosphere which they can judge from the degree of engagement, voluntary behaviors, and the emotions of a large group of students. After the discussion, the trainer emphasized the importance of the ability to recognize emotions of others in daily communication as well as in interactions in English class (e.g., teaching/learning effectiveness and positive relationship), and summarized different ways of recognizing emotions (e.g., facial expression, spatial movement, gesture, posture, pause, repetition, volume, pitch) supported by pictures or examples.

##### Generating positive emotions that facilitate thinking in week 3

In Week 3, the students were trained to improve their awareness and ability to generate positive emotions. They were asked to reflect on and discuss the following questions:

(1) What kind of positive emotions have you experienced in English learning?
(2) Are these emotions beneficial to you or your English studies?
(3) Do you frequently experience these emotions?
(4) Do you have the awareness to generate these kinds of emotions?
(5) If you experience these emotions, do you have the awareness to keep them.

After the discussion, the trainer summarized the types of positive emotions, the benefits they bring in different aspects including psychological, social, and psychical domains supported by PP theories, for instance, the broaden-and-build theory (Fredrickson, 1998, 2001, 2003) and the control-value theory of achievement emotions (Pekrun et al., 2007), empirical studies (Dewaele and Alfawzan, 2018; Saito et al., 2018; Li et al., 2019) and self-examples. The trainer also highlighted the need to enhance the awareness of generating and keeping positive emotions by employing some aspects of their TEI including self-motivation, trait optimism, and trait happiness based on the *trait EI model* (Petrides, 2009).

##### Understanding causes and consequences of emotions in self and others in week 4

In Week 4, students were trained to improve their emotional skills in identifying and analyzing the antecedents and consequences of emotions especially in English learning or use. First, students were encouraged to reflect on and share their emotional experiences. Then, two cases in English class based on the trainer’s own experience was provided for the students to identify the English teacher’s emotions, and the antecedents and consequences of them. Finally, the trainer summarized the students’ emotional experiences. Grounded in the broaden-and-build theory (Fredrickson, 1998, 2001, 2003), the control-value theory of achievement emotions (Pekrun et al., 2007), and some empirical studies (e.g., Li et al., 2018), the trainer pointed to the intra-and inter-personal factors for and process of the instigation of emotions, providing implications for effective emotion regulation by intervening on the antecedents of the emotional process. Furthermore, the trainer also pointed to the important role and possible consequences of emotions in both language learning and well-being, supported by the above-mentioned theories and empirical studies (e.g., Li et al., 2019).

##### Expressing emotions appropriately in week 5

Two cases were provided for students to improve their awareness of the importance of expressing emotions appropriately. The two cases based on the trainer’s own experience were both about students who benefited from expressing their emotions to someone they trust.

##### Regulating emotions in self and others effectively in week 6

Students were asked to reflect on the ways they regulate or manage their emotions and then to share them with their desk-mates. Some of them were invited to share the most effective way to cope with their negative emotions especially in English learning. The trainer summarized the most frequently mentioned emotional skills or strategies. Furthermore, according to the control-value theory (Pekrun et al., 2007), control and value appraisals of an emotional stimulus are prerequisites for emotion instigation. The trainer thus taught the students to change their appraisals of either control or value, further regulating their emotions fundamentally.

##### “Three activities” from PP

The present study adopted the PP-based “Three Activities” as part of the diary, which was developed in a language learning context by Gregersen et al. (2014) based on the original study of Seligman et al. (2005). “Three Activities” herein refers to “identifying “three good things, savoring positive experiences, and developing learned optimism” in English learning (Gregersen et al., 2014, p. 328, p. 340–345).

##### Semi-structured interview for students

Five students (who volunteered to participate when the class was informed of the interview) from the experiment class were interviewed after they completed the post-intervention questionnaire. The prompts for the interview were arranged as follows:

(1) What do you think of the AUGUER model of EI training? Beneficial or not? Why?
(2) Do you feel any changes in yourself and others during or after the training? If so, please give some examples.
(3) The program is over now, are you still willing to continue some emotional practices? Why?

##### Semi-structured interview for English teacher

The English teacher of the two classes was also interviewed. The prompts for the semi-structured interview were as follows:

(1) What do you think of the AUGUER model of EI training? Beneficial or not? Why?
(2) Do you feel any changes in the class? If so, what are they? Please specify them.

#### Procedures

A pre-test, treatment, and post-test design was adopted to testify the efficacy of EI intervention. First, the pre-intervention survey was conducted shortly after the quantitative results of Study 1 were obtained. More specifically, a pre-test between-group comparison was conducted between the two participating classes to guarantee that they were at comparable levels in terms of their TEI (*t* = −0.937, *p* = 0.351) and L2 classroom emotions, namely FLE (*t* = 1.060, *p* = 0.291) and FLA (*t* = 1.138, *p* = 0.258). Based on the results, the two classes were assigned as the intervention group and the control group, respectively.

Second, the EI intervention practice was implemented from June to July 2017. The intervention lasted for 6 weeks, consisting of ARGUER training of EI in evening classes (a prevalent form of self-study class in the evening for secondary school students in China), and weekday diary, reflection along ARGUER dimensions and “Three Activities” from PP.

Third, the post-intervention survey was conducted in the two groups using the same composite questionnaire as was used in the pre-intervention survey. Furthermore, five students from the intervention group and their English teacher participated in face-to-face semi-structured interviews.

### Results and Findings

ANCOVA tests for EI, FLE and FLA were conducted to compare intervention and control post-test scores with pre-test scores, controlled as co-variables. Table 6 displays the results.

**TABLE 6:** Pre- and post-comparison within the intervention and control groups.

	**Groups**		**Mean**	***N***	***SD***	**Skewness**	***SE1***	**Kurtosis**	***SE2***	***F*(1,105)**	***p***	**η*^2^***
EI	Intervention	TEI1	134.93	56	17.99	–0.017	0.319	–0.625	0.628	23.486	0.000	0.183
		TEI2	139.05	56	18.76	–0.181	0.319	–0.361	0.628			
	Control	TEI1	137.88	52	14.44	0.620	0.330	1.355	0.650			
		TEI2	138.37	52	14.45	0.630	0.330	1.207	0.650			
FLE	Intervention	FLE1	36.75	56	5.17	–0.060	0.319	0.914	0.628	6.346	0.013	0.057
		FLE2	38.98	56	5.60	–0.621	0.319	0.861	0.628			
	Control	FLE1	35.40	52	7.84	0.049	0.330	1.015	0.650			
		FLE2	36.06	52	7.33	–0.435	0.330	1.146	0.650			
FLA	Intervention	FLA1	98.27	56	19.24	0.262	0.319	0.723	0.628	17.726	0.000	0.144
		FLA2	95.63	56	17.55	–0.137	0.319	1.247	0.628			
	Control	FLA1	94.02	52	19.55	–0.092	0.330	0.015	0.650			
		FLA2	94.38	52	19.95	–0.233	0.330	0.231	0.650			

According to this table, in the intervention group, there was a significant increase in the scores for TEI and FLE, and a decrease in FLA, indicating the significant effect of EI training in all of the three variables. However, the effect size for the intervention on FLE was quite small. Altogether, we can conclude that the EI intervention consisting of ARGUER and the three activities is effective in improving EI and to correspondingly boost more positive emotions, reducing negative emotions in the context of EFL learning.

The qualitative interview with five students supported the efficacy of EI with rich details. They reported benefiting from the training in different aspects, most of which are inherently tied to the instigation of more positive emotions and reduced negative emotions. For example,

**Xiaoming** (pseudo name)

…I felt so surprised that we could have such kind of training. I am so **happy** that our headteacher cares about our mental states so much in addition to our learning achievements. The past 6 weeks were so **rewarding and unforgettable.** I felt much **more emotionally aware and competent**. Sometimes I felt so tired and frustrated In English class, I tried to **remind myself of the importance of English for my future, and I got motivated** to learn English again. Also, sometimes, when I felt bored because of the repeated recitation of English words, I would consciously **change my focus to other tasks which were more likely to bring me enjoyment and happiness…**

This extract showed the interviewee’s positive attitude toward EI training. It also indicates the benefits of the EI training including enhanced emotional awareness, increased self-motivation, improved awareness and the ability of emotion regulation, and more chances of experiencing positive emotions. Those outcomes are exactly the elements of EI according to its theoretical constructs (Petrides, 2009)., foregrounding the link between EI and emotions in English learning.

**Xiaoli** (pseudo name)

…Sometimes, when I saw that nobody responded to the enthusiastic English teacher, which was very **embarrassing**, I would volunteer to answer the question. I felt **the English teacher was relieved and very happy** about that, which also **made me happy**…

This extract indicated the interviewee’s positive change in responsive behaviors in class. She demonstrated an increased level of empathy toward the English teacher, which in turn brings her happiness. In other words, the EI element of empathy (Petrides, 2009) is linked to emotions.

**Xiaowang** (pseudo name)

…Time flies! The 6 weeks passed so quickly. I have become **accustomed to reflecting on my ARGUER and “three activities”** in my diary. I am not sure whether I will still keep the diary, but I am definitely sure that I will **make it a habit to reflect on my emotional skills and emotional experiences, not only in English learning but also in my daily life**. Thanks to the training, I find myself **stronger and happier**.

This extract indicted the effectiveness of the EI training in improving trait happiness and optimism, both of which are key elements of TEI construct (Petrides, 2009).

The above narrative corresponded to the two prompts for EI, highlighting the effectiveness and popularity of EI training. The English teacher, the leading side of classroom interaction, was also interviewed for her perceptions of students’ emotional states in English learning. The following excerpts are from her narrative:

…The training is **rewarding** for it points to **the mental health of students**, which is equally important for students’ development. However, so little has been done in this aspect. Thus, I hope you can do more in this field, which will help students become **emotionally stronger and healthier**, especially those introverts who dislike or feel uncomfortable expressing their emotions even when they need social support.

…The most impressive change is that I noticed **more responsive and motivated behaviors**. They seem to be more cooperative in class. Previously, it is easy to embarrass myself in a class activity where nobody reacts to me. Now, they seem to be **more considerate toward me**. Furthermore, their l**earning motivation is greatly enhanced**. Maybe, it is because you shared your motivating academic stories with them…

The teacher’s excerpts echoed students’ in the recognition of the benefits of the EI intervention: more empathy and enhanced language learning motivation. Considering the quantitative results and qualitative findings, the present study in the specific field of SLA, supports the main claim that increasing EI bolsters well-being, emotional experiences and social relationships in the studies by Nelis et al. (2011) and by Lea et al. (2018).

### Conclusions and Implications

Before concluding, we have to note two of the limitations in the present study. First, this study was a 6-week snapshot of EFL learners’ emotional experiences throughout the EI intervention. There is no way to know the lower limits and upper limits of the length of intervention implementation and assessment. Furthermore, it remains unknown whether the effectiveness from the intervention are short-lived or not. A delayed post-test could be a good way to elucidate this. Finally, most students from the two classes are children who have been left behind for many years and were studying in the boarding school when the study was conducted. The experiences of being left-behind without their parents supporting them daily may have some influence on their psychological features, including EI and emotional experiences in school (Liang and Wang, 2018). Thus, great caution should be taken in terms of the generalizability of the findings to samples or contexts.

Despite these limitations, this is the first mixed-method study showing an EI intervention program in EFL learners and its effectiveness in improving emotional skills as well as affecting emotional experiences based on the *trait EI theory*. This program was tightly grounded on the RULER approach in general psychology as well as “three activities” adapted from PP and used in a language education context (Gregersen et al., 2014). As we were trying to improve learners’ awareness of and skills in different EI dimensions, we were at the same time moving to increase their ability to cope with different emotion-related information in learning. As EI dimensions such as happiness, optimism, self-motivation, and emotional regulation can put the wind in one’s sails, more positive emotions can inevitably be boosted, and negative emotions can be reduced and managed because of the joint effects of gains in different EI dimensions and positive emotions.

The present study shows practical implications for L2 education in that it offers a theory-grounded and operational intervention practice from a PP perspective in the context of Chinese EFL learning.

## Summary of Study 1 and Study 2

To conclude, the current research revealed the significant relationships between TEI and L2 classroom emotions, presented a PP-based EI intervention practice in the EFL context, and testified to its effectiveness.

## Data Availability Statement

The datasets generated for this study are available on request to the corresponding author.

## Ethics Statement

Ethical review and approval was not required for the study on human participants in accordance with the local legislation and institutional requirements. All participants above the age of 16 provided written informed consent, For participants below the age of 16, written informed consent was obtained from their parents or their legal guardians.

## Author Contributions

CL designed the research, collected, processed, and analyzed the data, and wrote the whole manuscript. JX revised the manuscript.

## Conflict of Interest

The authors declare that the research was conducted in the absence of any commercial or financial relationships that could be construed as a potential conflict of interest.

## Appendix

**TABLE A1:** Outline of Emotional Intelligence Training Sessions.

**Session 1: Awareness of feelings and emotions in self and others.** 1. Welcome. 2. Introduction of the six sessions of EI training in class. 3. Introduction of “Three Activities” in the diary after class. 4. EI training. (1) Introduction of the definition, importance, malleability and training of EI in the context of L2 learning. (2) Introduction of the ARGUER model. (3) Dimension 1: A = Awareness of feelings and emotions in self and others. Inductive approach: Students’ reflection, discussion and sharing followed by the trainer’s (the first author) comments and summary supported by cases and examples. (4) Summary and homework (Reflection on Dimension 1 and diary based on “Three Activities”).
**Session 2: Recognizing emotions in self and others.** 1. Review of previous session. 2. EI training. Dimension 2: R = Recognizing emotions in self and others. (1) Improving students’ awareness to recognize the trainer’s emotions and emotion changes. (2) Identifying and recognizing the trainer’s emotions with verbal and non-verbal cues. 3. Summary and homework (Reflection on Dimension 2 and diary based on “Three Activities”).
**Session 3: Generating positive emotions that facilitate thinking.** 1. Review of previous session. 2. Dimension 3: G = Generating positive emotions that facilitate thinking. (1) Students’ reflection on their experiences of positive emotions in English learning and the benefits and frequencies of these experiences. (2) Students’ reflection on their awareness and competence of generating and keeping these positive emotions. (3) Case analysis. (4) Practice of positive thinking in some difficult situations in English learning. 3. Summary and homework (Reflection on Dimension 3 and diary based on “Three Activities”).
**Session 4: Understanding causes and consequences of emotions in self and others.** 1. Review of previous session. 2. Dimension 4: U = Understanding causes and consequences of emotions in self and others (1) Cases analyses. (2) Role play in given English teaching situations.
Summary and homework (Reflection on Dimension 4 and diary based on “Three Activities”).
**Session 5: Expressing emotions appropriately.** 1. Review of previous session. 2. Dimension 5: U = Expressing emotions appropriately. (1) Introduction of different ways of emotion expressions. (2) Case analysis. (3) Students’ anticipation of their emotion expression in future. 3. Summary and homework (Reflection on Dimension 5 and diary based on “Three Activities”).
**Session 6: Regulating emotions in self and others effectively.** 1. Review of previous session. 2. Dimension 6: R = Regulating emotions in self and others effectively. (1) Case analysis (2) Practice of positive thinking in some difficult situations in English learning. (3) Anxiety reduction practice. (4) Regulation of teachers’ emotion. Improving students’ awareness of noticing teachers’ emotions. Improving students’ awareness of being empathetic by analyzing the causes and consequences of teacher’s emotions. 3. Summary and homework (Reflection on Dimension 6 and the whole ARGUER model, and diary based on “Three Activities”).
